# Suicide in Ghana: How Could the Community-Based Health Planning and Service (CHPS) Effectively Contribute to Its Prevention?

**Published:** 2019-11

**Authors:** Abraham ASSAN, Moses AIKINS, Amirhossein TAKIAN

**Affiliations:** 1. Department of Health Management and Economics, School of Public Health, Tehran University of Medical Sciences, Tehran, Iran; 2. Global Policy and Advocacy Network (GLOOPLAN), Accra, Ghana; 3. School of Public Health, College of Health Science, University of Ghana, Legon, Ghana; 4. Department of Global Health and Public Policy, School of Public Health, Tehran University of Medical Sciences, Tehran, Iran; 5. Health Equity Research Center, Tehran University of Medical Sciences, Tehran, Iran

## Dear Editor-in-Chief

Suicide and suicide attempts are significant global public health challenge. Among the most common means of suicide worldwide are pesticide self-poisoning, hanging and firearm use. Regarding methods of suicide, studies have identified strong association between suicidal behavior and mental disorders –especially depression and alcohol use disorders. Further, many suicide cases happen spontaneously due to inabilities to handle life stress which emerge from financial difficulties, break-ups from relationships, chronic pain and illness or loss (1).

The health threat posed by suicide remains wide and severe – almost 800000 people die by suicide and many more attempts every year globally – remaining the second leading cause of death among 15–29-year-olds – majority (about 78%) occurring in low-and middle-income countries (LMICs) (1). In Ghana, about 1500 suicide cases are reported annually, and in each reported case of suicide are four unreported cases, summing the number of unreported cases to almost 6000 yearly (2).

According to WHO, “suicides are preventable with timely, evidence-based and often low-cost interventions”. However, little attention is given to addressing the phenomenon – only 28 countries worldwide report having national suicide prevention strategy (1). In Ghana, attempts to prevent suicide include the introduction of Ghana’s Criminal Code which criminalizes suicide and attempted suicide in the country. Nevertheless, considerable gaps continue to threaten suicide prevention. Following several policy debates, the Mental Health Authority of Ghana defines the growing suicidal rate in the country a “failure of the society” to provide supportive systems in addressing it (3). Moreover, there are compelling evidence supporting much flexibilities and successes from community-based multilevel interventions in combating suicide, especially in LMICs (4). The Community-based Health Planning and Services (CHPS) initiative emerged as the central tool to help “attain the goal of reaching every community with basic package of essential health services towards attaining universal health coverage and bridging the access inequity gap” – by promoting community involvements and ownership of program, in Ghana (5). Yet, general mental health services are mostly provided through specialized psychiatric hospitals or health centers, with relatively less government support to address the problem at the primary healthcare level. This paper presents three major ways CHPS can facilitate effective prevention of suicide in Ghana. They are: provision of life-course and multisectoral approach to addressing suicide; community participation; and provision of integrated surveillance and monitoring system. First, suicide is a multifaceted phenomenon and demands life-course and multisectoral approaches to its prevention and treatment (6). CHPS remain the key national policy that extend health services to the door-step of clients – rendering it appropriate within the Ghana Health Service (GHS) to liaise with other sectors (including education, labour, agricultural, legal, political, media), to promote comprehensive and integrated services delivery. Besides, we anticipate better integration of suicide prevention services into the service package of CHPS could serve as improved and cost-effective way of addressing the burden.

Second, whereas governments aim to take leading role in developing and implementing strategic suicide prevention strategies, local policymakers can help incorporate community needs into national plans (7). Since effective control of suicide requires greater leadership and community participation in service delivery (8), it is crucial that communities remain aware, accept interventions and contribute to the successes and continuity of programs through more meaningful engagement in the managements of public health initiatives. Hence, the community ought to have interest and participate in suicide prevention exercises. Otherwise, lack of perceived need by the community to fully fight suicide may undermine any prevention strategy (9). Again, CHPS is the only participatory body within the GHS that connect communities, health providers and other stakeholders, to help identify and solve societal health challenge.

Third, early detection, communication, treatments, and care for clients with mental and substance use disorders, chronic pain and acute emotional distress are critical in fighting suicide and attempted suicides (1). Yet, countries worldwide including Ghana are faced with the lack of reliable data on suicide (4). CHPS can effectively assist in the provision of accurate and nationally representative information on patterns, rates, characteristics and methods of suicides, and follow-up of vulnerable groups for referral and treatments (1).

Conclusively, the 2030 Sustainable Development Agenda addresses a new era for public health. Yet, suicide and suicide attempts impose substantial challenge to many countries. Appropriate integration of suicide prevention programs into community-based initiatives can, we envisage, serve as a dynamic window of opportunity to reimagine sustainable solutions to suicide in Ghana. Let alone, the role of the mass media, capacity building of community health professionals, and further studies into psychosocial causes and interventions to suicide could help achieve meaningful impacts.
